# Clinical characteristics and outcomes of antibiotic-associated encephalopathy in patients with end-stage kidney disease

**DOI:** 10.1080/0886022X.2022.2134025

**Published:** 2022-10-19

**Authors:** Qingxiu Huang, Jianbo Li, Naya Huang, Xi Xia, Yagui Qiu, Zhong Zhong, Zhenchuan Lin, Xiaowen Huang, Dihua Zhang, Fengxian Huang

**Affiliations:** a Department of Nephrology, The First Affiliated Hospital, Sun Yat-sen University, Guangzhou, China; b NHC Key Laboratory of Clinical Nephrology (Sun Yat-Sen University) and Guangdong Provincial Key Laboratory of Nephrology, Guangzhou, China; c Zhongshan Hospital of Traditional Chinese Medicine, Affiliated to Guangzhou University of Chinese Medicine, Zhongshan, China; d Department of Nephrology, Kidney and Urology Center, The Seventh Affiliated Hospital of Sun Yat-sen University, Shenzhen, China

**Keywords:** Antibiotic-associated encephalopathy, end-stage kidney disease, prevalence, risk factors, outcomes

## Abstract

**Objective:**

End-stage kidney disease (ESKD) patients have a higher risk of antibiotic-associated encephalopathy (AAE) than other patients. We aimed to evaluate the prevalence, risk factors and outcomes of AAE in ESKD patients.

**Method:**

A retrospective study of ESKD patients treated with intravenous antibiotics in our hospital from Jan. 1, 2006, to Dec. 31, 2015 was performed. AAE was diagnosed by the modified Delphi method. Control individuals were randomly selected from the remaining patients who did not exhibit neurologic symptoms. Logistic regression analysis was used to identify risk factors for AAE as well as the association between AAE and outcome.

**Result:**

A total of 2104 patients were included in the study. The prevalence of AAE in our study was 4.4% (92/2104). The multivariate logistic regression analysis revealed that anuria (OR = 8.04, 95% CI: 4.13–15.65, *p* < 0.001), history of central nervous system disorder (OR = 3.03, 95% CI: 1.21–7.56, *p* = 0.018) and hypoalbuminemia (OR= 1.87, 95% CI: 1.01–3.47, *p* = 0.046) were independent factors associated with AAE in ESKD patients. After adjustment for confounders, AAE was associated with composite outcomes of in-hospital mortality and treatment withdrawal (OR = 4.36, 95% CI: 2.09–9.10, *p* < 0.001).

**Conclusion:**

The prevalence of AAE was 4.4% in ESKD patients and varied among different antibiotics. Anuria, history of central nervous system disorder and hypoalbuminemia were associated with AAE in ESKD patients. AAE is associated with worse outcomes in ESKD patients.

## 1. Introduction

Antibiotics are commonly prescribed to end-stage kidney disease (ESKD) patients since they are vulnerable to a variety of infections, including pneumonia, peritonitis and catheter-related infections [1]. Most antibiotics are predominantly or partially excreted by the kidneys. ESKD patients treated with antibiotics without dose adjustment are at risk of developing side effects, including encephalopathy [2]. Previous studies have shown that encephalopathy can increase the length of hospital stay [3,4], the cost of medical expenses [5], the presence of in-hospital complications [6], and the 1-year mortality rate [7].

Antibiotic-associated encephalopathy (AAE) is an etiology of encephalopathy that is often overlooked but can lead to serious consequences [2]. It manifests as impaired consciousness, perception or memory; bizarre behavior; or convulsion after antibiotic accumulation [8]. The AAE rate has been reported as 0.1–1% in previous studies [9,10]. However, in clinical practice, we have noted that the occurence of AAE may be underestimated in ESKD patients. Previous studies on AAE were limited to case reports or small series, and few studies have focused on the clinical features and prevalence of AAE among ESKD patients [8, 10–14]. We speculate that some factors are associated with AAE and that AAE is associated with poor outcomes. In this case–control study, we aimed to evaluate the prevalence and features of AAE among ESKD patients and further explore the associated factors and outcomes of AAE.

## 2. Methods

### 2.1. Study participants

The study was approved by the review board of The First Affiliated Hospital of Sun Yat-sen University (approval NO. [2016]215) and complied with the guidelines of the Declaration of Helsinki (2013 Amendment). The requirement for informed consent was waived because the study was retrospective.

The retrospective study was performed and included ESKD patients treated with antibiotics at The First Affiliated Hospital of Sun Yat-sen University. The data of 2898 in-hospital ESKD patients diagnosed with infection in our center from Jan. 1, 2006, to Dec. 31, 2015, were retrieved from our medical record system. Patients who received any intravenous antibiotics during hospitalization and who were older than 14 years old were included in the study. Those who exhibited neuropsychiatric abnormalities before antibiotic administration and did not have complete data were excluded.

**Figure 1.**
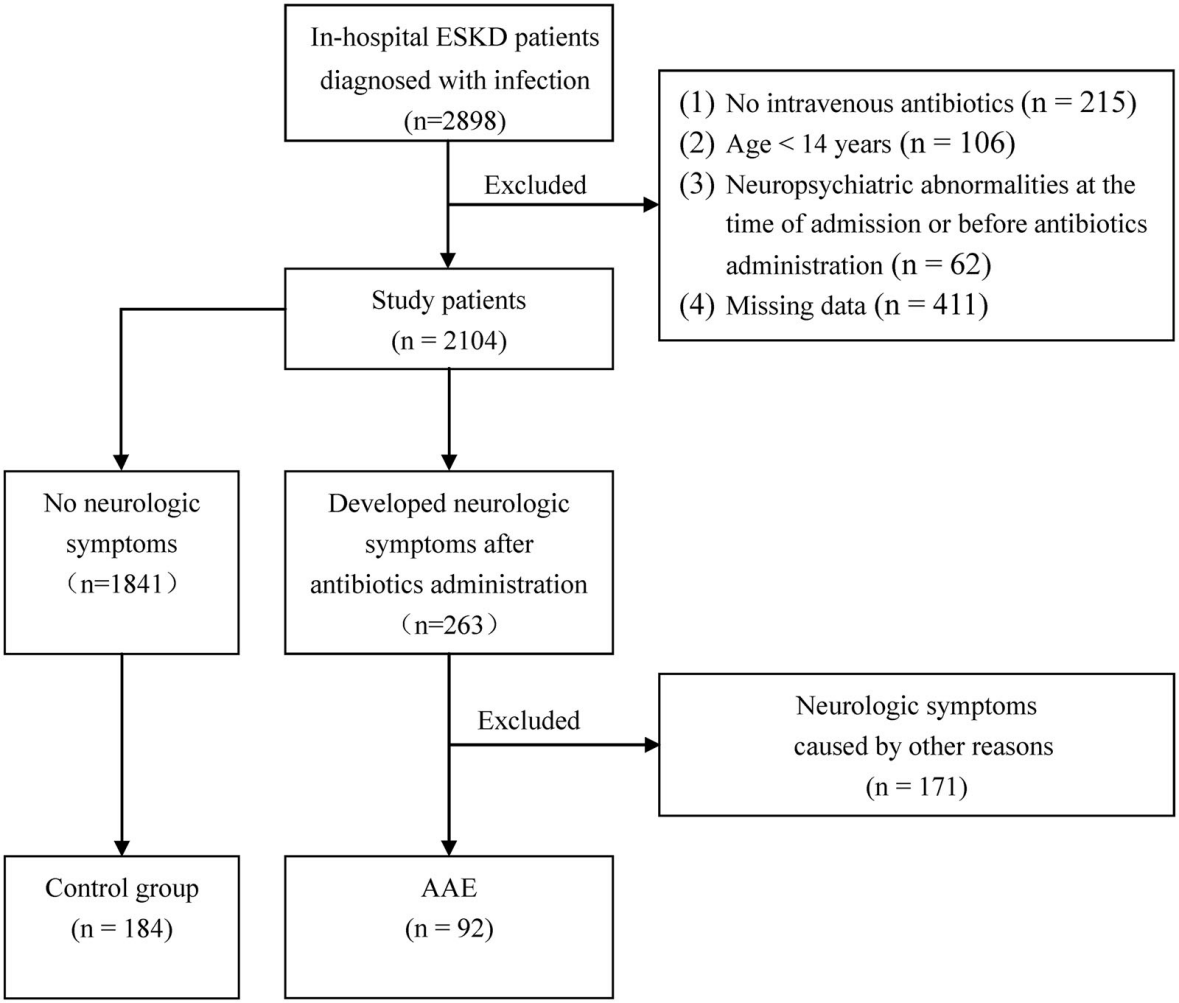
Study workflow. AAE: antibiotic-associated encephalopathy; ESKD: end-stage kidney disease.

A total of 2104 patients were included in the study to explore the prevalence of AAE. The medical records of these 2104 patients were reviewed to determine whether they exhibited neurologic symptoms subsequent to the administration of intravenous antibiotics. Among them, 263 patients developed neurologic symptoms after the initiation of antibiotics, of whom 92 patients developed encephalopathy that was attributed to antibiotic use. To evaluate the associated factors and outcomes, another 184 patients were randomly selected as the control group from the remaining 1841 patients who did not develop neurologic symptoms after the administration of antibiotics (Figure 1).

### 2.2. Clinical data collection

Data regarding AAE symptoms, signs and clinical outcomes were collected. Data regarding demographic information, cause of ESKD, dialysis modality, dialysis vintage, urine volume, infection type, antibiotic category and coadministered drugs, biochemical parameters on the first day of antibiotic administration and comorbidities (including cardiovascular disease, history of hepatic disease and history of central nervous system disorder) were collected.

ESKD was defined as an estimated glomerular filtration rate (GFR) of ≤15 mL/min/1.73 m^2^ and included both CKD5 and CKD5D. Cardiovascular disease (CVD) was defined as a history of angina pectoris, myocardial infarction, angioplasty, coronary artery bypass or heart failure. A history of hepatic disease was defined as a history of hepatitis, liver cirrhosis and liver cancer. A history of central nervous system disorder was defined as a history of stroke, encephalorrhagia, intracranial infection and epilepsy. Hypoalbuminemia was defined as serum albumin < 35 g/L.

### 2.3. Diagnosis of antibiotic-associated encephalopathy

AAE was diagnosed according to the criteria and methods of the Mayo Center [15] as follows: 1) encephalopathy, including one or more of the following manifestations: change in consciousness, myoclonus, seizure, hallucinations, delusions, lack of coherent speech; and 2) a clear temporal relationship between encephalopathy and antibiotic administration (encephalopathy appeared after the initiation of medication, persisted or worsened during medication administration, and improved or resolved after drug discontinuation). Patients with encephalopathy caused by other reasons, such as cerebrovascular attack, nervous system infection, demyelinating disease, dialysis imbalance, neuropsychiatric lupus, etc., were excluded. The modified Delphi method was used to determine the likelihood of causality between encephalopathy and antibiotic use. All possible cases were reviewed by five coauthors (HQX, LJB, HFX, ZDH, HNY) independently. The diagnosis of AAE was made if three or more of the reviewers agreed that encephalopathy was attributable to antibiotic use.

### 2.4. Statistical analysis

Statistical analysis was performed with SPSS 22.0 statistical software. Results with a two-sided P value of < 0.05 were considered statistically significant.

All values are expressed as the means ± SDs (standard deviations) or medians [25th, 75th percentiles] for continuous variables and frequencies for categorical variables. The Shapiro–Wilk test was used to assess normality. For comparisons of continuous variables, we used the t test or the Wilcoxon rank sum test as appropriate. Comparisons between subgroups with categorical variables were performed with the chi-square test.

All variables were examined by univariate analysis to identify risk factors for AAE, and factors with P values of <0.10 in the univariate analysis were included as candidate predictors in the multivariable logistic regression model by forward stepwise regression. Multivariate logistic regression analysis was performed to determine the factors that were independently associated with AAE. The performance of the multivariate logistic regression model was evaluated using the receiver operating characteristic (ROC) curve.

Univariate and multivariate logistic regression analyses were performed to determine the association between AAE and outcomes. Because of local customs, some seriously ill patients preferred to pass away at home rather than in hospital. Thus, composite outcomes of in-hospital mortality and treatment withdrawal were refer to bad outcome in this study.

## 3. Results

### 3.1. Prevalence and clinical manifestations of AAE in the total study population

A total of 2104 patients were included in the study, of whom 263 developed neurologic symptoms after the initiation of antibiotics. Finally, a total of 92 cases fulfilled the AAE diagnostic criteria (Figure 1). The prevalence of AAE in ESKD in hospital patients was 4.4%.

The neurological symptoms of AAE were diverse and are shown in Table 1. The common symptoms were delirium, bizarre behavior, myoclonus and convulsions. The above symptoms often changed as the disease progressed in a patient; that is, patients often progressed from confusion to delirium, from mania to drowsiness, or from delusions to convulsions. None of the 92 patients exhibited typical signs of nervous system localization, meningeal irritation, or ataxia.

**Table 1.** The neurologic symptoms of antibiotic-associated encephalopathy.

Neurologic symptoms	n	Percentages (%)
Depressed level of consciousness	14	18.2
Delirium	29	31.5
Confusion	18	19.6
Bizarre behavior	24	26.1
Myoclonus or tremor	23	25.0
Seizure	31	33.7
Insomnia	12	13.0

A depressed level of consciousness was defined as the presence of somnolence, sopor or coma. Delirium was defined as a state of violent mental agitation often accompanied by hallucinations or delusions. Confusion was characterized by a lack of clear and orderly thought and behavior and unresponsiveness.

**Figure 2.**
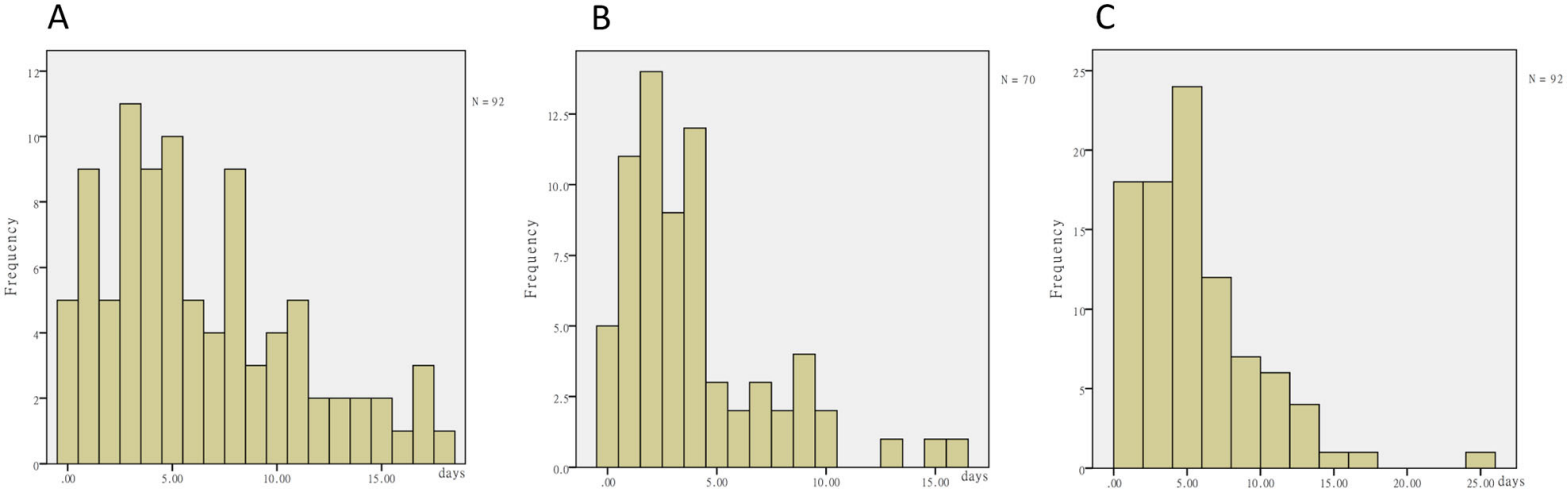
The frequency distribution histogram of the duration of antibiotic treatment or encephalopathy. (A) The duration from antibiotic initiation to the occurrence of antibiotic-associated encephalopathy. (B) The duration from antibiotic discontinuation to the improvement of antibiotic-associated encephalopathy. (C) The duration of antibiotic-associated encephalopathy.

Neurological symptoms began at median day 5 (3, 9) after antibiotic initiation (Figure 2(A)) and improved or resolved at median day 3 (2, 5) after the antibiotic was discontinued or adjusted (Figure 2(B)). The mean duration of encephalopathy was 5 (2.5, 7) days, while the longest duration was 25 days (Figure 2(C)).

### 3.2. Prevalence and clinical manifestations of AAE among different antibiotic drug classes

The AAE rates of different antibiotics in ESKD patients are shown in Table 2. Ceftazidime, cefoperazone, levofloxacin and imipenem use were associated with the highest numbers of AAE cases. However, the AAE rates observed following the use of lomefloxacin, ceftazidime, imipenem and cefepime ranked at the top. It should be noted that ten cases diagnosed with AAE were caused by a combination of two types of antibiotics.

**Table 2.** The AAE rates of different antibiotics in ESRD patients.

Antibiotic	No. of AAE cases^a^	No. of patients receiving antibiotics^b^	AAE rates
Cephalosporins			
Ceftazidime	37	473	7.82%
Cefoperazone	12	559	2.15%
Ceftriaxone	2	213	0.94%
Latamoxef	3	143	2.10%
Cefepime	2	28	7.14%
Carbapenems			
Imipenem	11	149	7.38%
Meropenem	1	110	0.91%
Panipenem	2	36	5.56%
Quinolones			
Moxifloxacin	8	410	1.95%
Levofloxacin	12	400	3.00%
Ciprofloxacin	1	115	0.87%
Lomefloxacin	1	8	12.50%
Others			
Piperacillin	1	250	0.40%
Penicillin	1	32	3.13%
Fluconazole	1	115	0.87%
Voriconazole	1	39	2.56%
Caspofungin	1	32	3.13%
Vancomycin	2	63	3.17%
Teicoplanin	1	80	1.25%
Linezolid	1	46	2.17%
Azithromycin	1	156	0.64%
Total study patients	92	2104	4.37%

AAE: antibiotic-associated encephalopathy; ESRD: end-stage renal disease; NO.: number.

^a^There are ten cases diagnosed with AAE were caused by a combination of two types of antibiotics.

^b^Patients who were administered more than two types of antibiotics at the same time or consecutively were counted repeatedly.

Further statistical analysis was performed to compare the AAE rates of different antibiotics within the same class. Among the cephalosporins, the AAE rate following ceftazidime use was significantly higher than that following the use of cefoperazone or ceftriaxone (ceftazidime vs. cefoperazone: (7.8% (37/473) vs. 2.1% (12/559), *p* < 0.001; ceftazidime vs. ceftriaxone: (7.8% (37/473) vs. 0.9% (2/213), *p* < 0.001)). Among the carbapenems, the AAE rate following the use of imipenem was significantly higher than that following the use of meropenem (7.4% (11/149) vs. 0.9% (1/110), *p* = 0.015). There was no significant difference in the AAE rates among quinolones, including moxifloxacin (8/410, 2.0%), levofloxacin (12/400, 3.0%) and ciprofloxacin (1/115, 0.9%) (*p* = 0.278).

The main symptoms of neurotoxicity induced by different antibiotics were different. Cephalosporin and quinolone antibiotic-associated neurotoxicity manifested more often as mental disorders, and carbapenem neurotoxicity manifested more often as convulsions (Table 3).

**Table 3.** Main neurologic symptoms across different antibiotic drug classes.

Main neurologic symptoms	Cephalosporin (*n* = 51)	Quinolone (*n* = 13)	Carbapenem (*n* = 9)	*P* value
Mental disorders	39 (74.6%)	13 (100%)	4 (44.4%)	0.004
Myoclonus or tremor	14 (27.5%)	2 (15.4%)	1 (11.1%)	0.396
Convulsions	14 (27.5%)	3 (23.1%)	5 (55.6%)	0.223

### 3.3. Risk factors for AAE

To evaluate the associated factors and outcomes, another 184 patients were randomly selected as the control group from the remaining 1841 patients who did not develop neurologic symptoms after the administration of antibiotics. The demographic and clinical characteristics of the 92 ESKD patients with AAE and 184 patients without AAE are summarized in Table 4. The AAE group was older than the non-AAE group (59.8 ± 17.1 vs. 50.9 ± 18.6, *p* < 0.001) and had a longer hospital stay [22.5 (12, 34) vs. 15 (8, 21), *p* < 0.001] and a lower proportion of hospitalization in the nephrology ward [50 (54.3%) vs. 130 (70.7%), *p* = 0.007]. Furthermore, patients with AAE had a longer duration of dialysis; a higher rate of anuria; and higher rates of cardiovascular disease, history of central nervous system disorder, pneumonia, aspartate aminotransferase (AST), and hypoalbuminemia than those without AAE.

**Table 4.** Baseline characteristics of ESRD patients with and without AAE.

Characteristics	AAE (*n* = 92)	Non-AAE (*n* = 184)	*p* Value
Age (years)	59.8 ± 17.1	50.9 ± 18.6	**<0.001**
Male (%)	54 (58.7)	111 (60.3)	0.698
Hospital stay (days)	22.5 (12, 34)	15 (8, 21)	**<0.001**
Hospitalized in nephrology ward (%)	50 (54.3)	130 (70.7)	**0.011**
Cause of ESRD (%)			
Glomerulonephritis	33 (35.9)	76 (41.3)	0.165
Diabetic kidney disease	20 (21.7)	32 (17.4)	
Benign nephrosclerosis	12 (13.0)	17 (9.2)	
Lupus nephritis	9 (9.8)	8 (4.3)	
Other	18 (19.6)	51 (27.7)	
RRT (%)			0.220
No RRT	8 (8.7)	28 (15.2)	
Hemodialysis	47 (51.1)	96 (52.2)	
Peritoneal dialysis	37 (40.2)	60 (32.6)	
Dialysis vintage (months)	12.0 (0.3,36.0)	0.51 (0.21, 6.90)	**<0.001**
Anuria (%)	51 (55.4)	22 (12.0)	**<0.001**
Hypertension (%)	76 (82.6)	160（87.0)	0.333
Cardiovascular disease (%)	22 (23.9)	21 (11.4)	**0.007**
History of hepatic disease (%)	10 (10.9)	21 (11.4)	0.893
History of central nervous system disorder (%)	22 (23.9)	10 (5.4)	**<0.001**
Infection type (%)			
Pneumonia	72 (78.3)	107 (58.2)	**0.001**
Peritonitis	8 (8.7)	13 (7.1)	0.630
Bacteremia	3 (3.3)	12 (6.5)	0.260
Urinary tract infection	8 (8.7)	28 (15.2)	0.129
Laboratory tests			
WBC (10^9^/L)	7.3 (5.2, 10.3)	7.9 (5.9, 9.6)	0.693
NEU (%)	0.8 (0.7, 0.8)	0.7 (0.6, 0.8)	0.055
Hemoglobin (g/L)	79.0 (71.0, 90.0)	84.0 (72.0, 98.0)	0.080
Creatinine (mmol/L)	720.5 (557.5, 951.2)	748.0 (578, 948)	0.691
BUN (umol/L)	25.3 (21.5, 30.0)	25.0 (21.0,30.0)	0.669
Albumin (g/L)	29.0 (26.0, 33.0)	33.0 (28.9, 37.0)	**<0.001**
Hypoalbuminemia (n%)	74 (80.4)	103 (56.0)	<0.001
ALT (U/L)	15.0 (5.0, 30.0)	19.0 (12.0, 27.0)	0.065
AST (U/L)	20.0 (13.3, 30.8)	17.0 (12.0, 26.0)	**0.031**
TBIL (umol/L)	6.75 (4.5, 12.2)	6.4 (4.2, 10.1)	0.435

AAE: antibiotic-associated encephalopathy; ESRD: end-stage renal disease; RRT: renal replacement therapy; CVD: cardiovascular disease; WBC: white blood cell; NEU: neutrophil proportion; BUN: blood urine nitrogen; ALT: alanine aminotransferase; AST: aspartate aminotransferase; TBIL: total bilirubin. Hypoalbuminemia was defined as serum albumin < 35 g/L. Bold values indicates significant statistical differences.

The univariate logistic regression analysis showed that age (OR = 1.03, *p* < 0.001); dialysis vintage (OR = 1.01, *p* = 0.003); anuria (OR = 9.16, *p* < 0.001); CVD (OR = 2.44, *p* = 0.008); history of central nervous system disorder (OR = 5.47, *p* < 0.001) and pneumonia (OR = 2.59, *p* = 0.001); hypoalbuminemia (OR = 3.23, *p* < 0.001); and AST level (OR = 1.01, *p* = 0.033) were related to the onset of AAE in ESKD patients (Table 5).

**Table 5.** Univariable and multivariable logistic analyses of factors associated with AAE in ESRD patients.

Variable	Univariate logistic analysis		Multivariable logistic analysis	
	OR (95% CI)	*p* Value	OR (95% CI)	*p* Value
Age (per 1 yr)	1.03 (1.01, 1.04)	**<0.001**	1.02 (1.00, 1.03)	0.053
Sex, men versus women	0.94 (0.56, 1.56)	0.795		
Cause of ESRD (%)				
Glomerulonephritis	ref.	–		
Diabetic kidney disease	1.44 (0.72, 2.88)	0.302		
Benign nephrosclerosis	1.63 (0.70, 3.78)	0.259		
Lupus nephritis	2.59 (0.92, 7.30)	0.072		
Other	0.81 (0.41, 1.60)	0.547		
RRT (%)				
No RRT	ref.			
Hemodialysis	1.71 (0.73, 4.05)	0.220		
Peritoneal Dialysis	2.16 (0.89, 5.33)	0.089		
Dialysis vintage(months)	1.01 (1.00, 1.02)	**0.003**		
Anuria (%)	9.16 (5.00, 16.90)	**<0.001**	8.04 (4.13, 15.65)	**<0.001**
Complications (%)				
CVD	2.44 (1.26, 4.72)	**0.008**		
History of hepatic disease	0.95 (0.43, 2.10)	0.893		
History of central nervous system disorder	5.47 (2.46, 12.14)	**<0.001**	3.03 (1.21, 7.56)	**0.018**
Infection type (%)				
Pneumonia	2.59 (1.46, 4.61)	**0.001**		
Peritonitis	1.25 (0.50, 3.14)	0.631		
Bacteremia	0.48 (0.13, 1.76)	0.269		
Urinary tract infection	0.53 (0.23, 1.217)	0.134		
Laboratory tests				
WBC (per 1.0 × 10^9^/L)	1.01 (0.94, 1.08)	0.784		
NEU (per 1%)	3.69 (0.47, 28.99)	0.214		
Hemoglobin (per 1.0 × g/L)	0.99 (0.97, 1.00)	0.059	0.97 (0.96, 1.00)	0.052
Hypoalbuminemia	3.23 (1.79, 5.84)	**<0.001**	1.87 (1.01, 3.47)	**0.046**
ALT (per 1.0 × U/L)	1.00 (0.99, 1.01)	0.495		
AST (per 1.0 × U/L)	1.01 (1.00, 1.02)	**0.033**		
TBIL (per 1.0 × umol/L)	1.01 (1.00, 1.03)	0.138		

AAE: antibiotic-associated encephalopathy; ESRD: end-stage renal disease; RRT: renal replacement therapy; WBC: white blood cell; NEU: neutrophil proportion; BUN: blood urine nitrogen; ALT: alanine aminotransferase; AST: aspartate aminotransferase; TBIL: total bilirubin. Hypoalbuminemia was defined as serum albumin < 35 g/L. Bold values indicates significant statistical differences.

**Figure 3.**
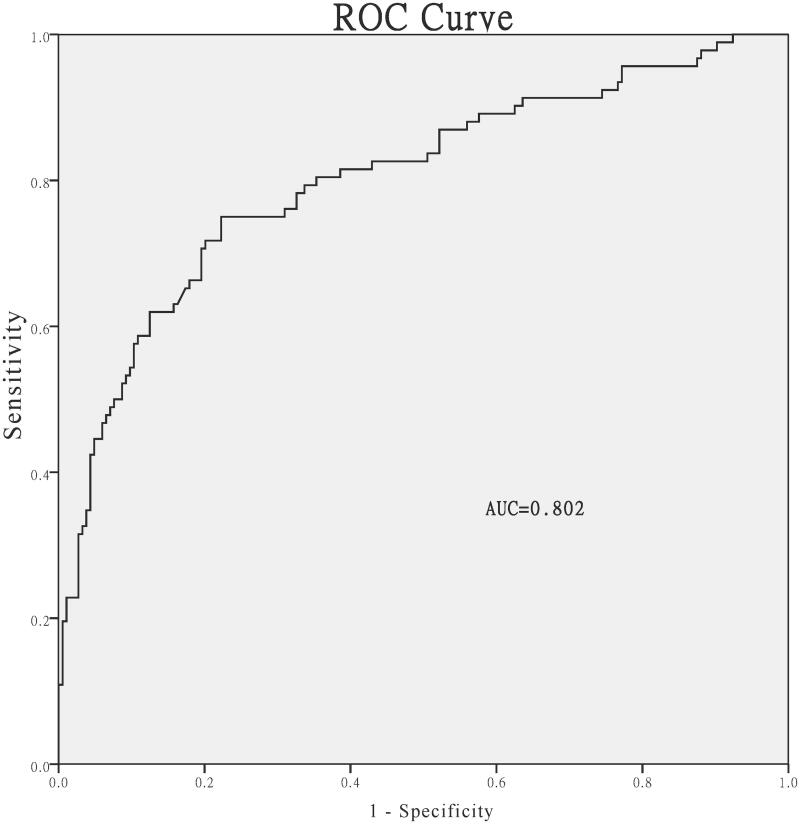
Receiver operating characteristic curve for the multivariate logistic regression model.

All variables with a P value of <0.10 in the univariable analyses were included as candidate predictors in the multivariable logistic regression model by forward stepwise regression. We identified anuria (OR = 8.04, 95% CI: 4.13–15.65, *p* < 0.001), history of central nervous system disorder (OR = 3.03, 95% CI: 1.21–7.56, *p* = 0.018) and hypoalbuminemia (OR= 1.87, 95% CI: 1.01–3.47, *p* = 0.046) as independent factors associated with AAE (Table 5). We plotted the ROC curve to assess the performance of the multivariate logistic regression model. The area under the ROC curve (AUC) was 0.802 (Figure 3).

### 3.4. Outcomes of AAE

The outcomes in the AAE group were worse than those in the non-AAE group, as shown in Table 6. Among 92 AAE patients, 28 patients (30.4%) died in the hospital, and 13 patients (14.1%) withdrawed treatment because of poor outcomes and high costs. One of the patients died of aspiration asphyxia caused by a convulsion.

**Table 6.** Outcome of ESRD patients with and without AAE.

Outcome	AAE	Non-AAE	*p* Value
Recover	51 (55.4%)	157 (85.3%)	<0.001
Died in hospital	28 (30.4%)	5 (2.7%)	
Treatment withdrawal	13 (14.1%)	23 (12.5%)	

AAE: antibiotic-associated encephalopathy.

The association between AAE and composite outcomes of in-hospital mortality and treatment withdrawal with defined models are listed in Table 7. Regardless of the adjustment method used, AAE was significantly associated with composite outcomes of in-hospital mortality and treatment withdrawal. After adjusting for sex, age, anuria, cardiovascular disease, hypoxemia, diabetes, history of hepatic disease, history of central nervous system disorder, infection site, levels of serum albumin, hemoglobin levels, serum creatinine levels, and serum bilirubin levels, AAE showed a stronger association with composite outcomes of in-hospital mortality and treatment withdrawal (OR = 4.36, 95% CI: 2.09–9.10; *p* < 0.001).

**Table 7.** Associations of AAE with composite outcomes of in-hospital mortality and treatment withdrawal.

Model	OR (95% CI)	*p* Value
Unadjusted	4.50 (2.52, 7.96)	<0.001
Model 1	4.09 (2.26, 7.37)	<0.001
Model 2	4.21 (2.18, 8.12)	<0.001
Model 3	4.16 (2.13, 8.14)	<0.001
Model 4	4.61 (2.30, 9.27)	<0.001
Model 5	4.36 (2.09, 9.10)	<0.001

CI: confidence interval; AAE: antibiotic-associated encephalopathy; OR: odds ratio.

Model 1: adjusted for age and sex.

Model 2: adjusted for model 1 and anuria.

Model 3: adjusted for model 2 and cardiovascular disease, hypoxemia, diabetes, liver disease, and nervous system disease.

Model 4: adjusted for model 3 and infection site.

Model 5: adjusted for model 4 and levels of serum albumin, hemoglobin, serum creatinine and serum bilirubin.

## 4. Discussion

This retrospective study was conducted to evaluate AAE in ESKD patients. The prevalence of AAE in ESKD patients was 4.4%. As expected, different antibiotics exhibited different AAE rates. In addition, anuria, history of central nervous system disorder and hypoalbuminemia were associated with AAE.

Neurotoxicity is described as a rare side effect of most antibiotic drugs, with a rate of occurrence of less than 0.1% [9]. However, our findings showed that the prevalence of AAE in ESKD patients was 4.4%. According to previous literature, the high prevalence of AAE in ESKD patients may be related to the following factors [16–18]. First, most antibiotics are metabolized in the kidneys. Without an appropriate reduction in drug dose according to the creatinine clearance rate, these antibiotics have a prolonged half-life in patients with a reduced glomerular filtration rate (GFR) and tend to accumulate in the body. Second, patients with chronic kidney failure often have hypoalbuminemia and internal environmental disorders. Consequently, the concentration of antibiotics bound to albumin is reduced, resulting in a relatively high concentration of free and active antibacterial compounds [19]. In addition, some scholars speculate that the blood–brain barrier and brain permeability of patients with chronic kidney failure are impaired [20].

Our research found that there were differences in the AAE rates of different antibiotics. To our knowledge, this is the first study to investigate the AAE rate of different antibiotics. Our findings have certain value for the selection of antibiotic regimens in ESKD patients. Cefepime neurotoxicity is widely reported [16,17], and the United States Food and Drug Administration (FDA) released a safety announcement in 2012 that indicated that cefepime should be administered with caution in patients with kidney failure [2]. Our study showed that the AAE rate following cefepime use was 7.14%, which was consistent with previous studies [16]. In addition, cefepime is not widely used in our center since cefepime neurotoxicity is widely known to occur. Only 2 of 92 AAE cases were related to cefepime use in the study. We suggest that other antibiotics with a high risk of neurotoxicity should be administered with cautious and appropriate dose adjustments in ESKD patients too.

Previous studies have shown that the difference in neurotoxicity risk may be related to the drug structure group, drug metabolism pathway and ability to penetrate the blood–brain barrier [8]. First, the encephalopathy rate may be related to the structure of the drug. Previous studies have shown that the neurotoxicity of cephalosporins and carbapenems is related to the amino structure of the side chain of the β-lactam ring C-2, which can inhibit the binding of neuronal γ-aminobutyric acid (GABA) receptors to GABA, thereby enabling neuronal activity enhancement and predisposing the patient to irritability and incoherent speech. All β-lactam drugs have the possibility of inducing convulsions and mental disorders, but not every β-lactam has the same binding affinity to GABA receptors [21,22]. Quinolone antibiotics can directly inhibit the effect of GABA. Bioorg’s animal experiments confirmed that the side groups of different quinolone drugs affected their GABA receptor affinity differently [23,24]. Some trials even attempted to modify the side group structure of norfloxacin to increase its affinity to GABA receptors so that such quinolone antibiotics could be used as new anxiolytics [25]. Moreover, the structure of the drug may be related to different main neurologic symptoms; for example, quinolone antibiotic neurotoxicity manifested more often as mental disorders, and carbapenem neurotoxicity manifested more often as convulsions. Second, neurotoxicity may be related to the drug metabolism pathway. Our study found that the AAE rate following ceftazidime use was significantly higher than that following the use of cefoperazone and ceftriaxone. The possible explanation is that ceftazidime is predominantly excreted *via* the kidneys, while cefoperazone and ceftriaxone are predominantly excreted *via* bile. Therefore, ceftazidime is more likely to accumulate in ESKD patients than the other two third-generation cephalosporin antibiotics.

Shamik summarized the case reports published thus far and suggested that age, renal function, and a history of neurological disease may be risk factors for AAE [8]. However, comparison with a control group was lacking, and this conclusion has not yet been confirmed by studies with higher levels of evidence. This retrospective case–control study showed that anuria, history of central nervous system disorder and hypoalbuminemia may be risk factors for AAE. Anuria is equivalent to no residual kidney function in a sense [26]. Limited by the experimental conditions and the data obtained, the residual renal function was not calculated for all individuals in this study; therefore, "anuria" was used as a proxy for "no residual renal function". Previous studies have shown that residual renal function has an impact on pharmacokinetics in patients who have undergone renal replacement therapy [19]. Thus, the International Society of Peritoneal Dialysis (ISPD) guidelines recommend that the dosage of intraperitoneal antibiotic therapy should be adjusted according to the presence and absence of urine in the treatment of peritonitis [27]. We speculate that a possible reason for the influence of hypoalbuminemia is that hypoalbuminemia increases the concentration of biologically available antibiotic compounds; moreover, hypoalbuminemia is related to poor nutritional status, decreased immune function, and severe infection [28]. Overall, based on previous literature and our research [8–10], it is recommended that the use of neurotoxic antibiotics is avoided as much as possible in patients with old age, neurological diseases, hypoalbuminemia, and anuria. For patients without residual renal function, it is recommended that the antibiotic dosage is adjusted strictly according to renal function parameters.

Previous AAE studies comprise mainly case reports and reviews. For the first time, we explored the risk factors for AAE in a case–control study. However, there are limitations to our study. First, this study was retrospective in nature. Second, the reported analyses were limited to data from a single center, and the sample size was relatively small. Third, there is no gold standard for the diagnosis of AAE. Finally, the impact of the antibiotic drug dosage could not be assessed since this study did not focus on a specific antibiotic. Higher-level prospective studies should be conducted in the future.

## 5. Conclusion

The prevalence of AAE was 4.4% in ESKD patients and varied among different "culprit" antibiotics. The factors associated with AAE in ESKD patients were anuria, history of central nervous system disorder, and hypoalbuminemia. AAE is associated with worse outcomes in ESKD patients. Thus, ESKD patients, especially those with anuria, hypoproteinemia or a history of central nervous system disorder, should be cautiously prescribed antibiotics with a low risk of neurotoxicity.

## Data Availability

The datasets used and/or analyzed during the current study are available from the first author on reasonable request.

## References

[1] Dalrymple LS, Go AS. Epidemiology of acute infections among patients with chronic kidney disease. Clin J Am Soc Nephrol. 2008;3(5):1487–1493. 18650409 10.2215/CJN.01290308 PMC4571152

[2] Sonck J, Laureys G, Verbeelen D. The neurotoxicity and safety of treatment with cefepime in patients with renal failure. Nephrol Dial Transplant. 2008;23(3):966–970. 18175786 10.1093/ndt/gfm713

[3] Ely EW, Shintani A, Truman B, et al. Delirium as a predictor of mortality in mechanically ventilated patients in the intensive care unit. JAMA. 2004;291(14):1753–1762. 15082703 10.1001/jama.291.14.1753

[4] Pompei P, Foreman M, Rudberg MA, et al. Delirium in hospitalized older persons: outcomes and predictors. J Am Geriatr Soc. 1994;42(8):809–815. 8046190 10.1111/j.1532-5415.1994.tb06551.x

[5] Covinsky KE, Justice AC, Rosenthal GE, et al. Measuring prognosis and case mix in hospitalized elders. The importance of functional status. J Gen Intern Med. 1997;12(4):203–208. 9127223 10.1046/j.1525-1497.1997.012004203.x PMC1497092

[6] Girard TD, Pandharipande PP, Ely EW. Delirium in the intensive care unit. Crit Care. 2008;12(Suppl 3):S3. 10.1186/cc6149 PMC2391269 18495054

[7] Leslie DL, Zhang Y, Holford TR, et al. Premature death associated with delirium at 1-year follow-up. Arch Intern Med. 2005;165(14):1657–1662. 16043686 10.1001/archinte.165.14.1657

[8] Bhattacharyya S, Darby RR, Raibagkar P, et al. Antibiotic-associated encephalopathy. Neurology. 2016;86(10):963–971. 26888997 10.1212/WNL.0000000000002455

[9] Owens RC, Jr., Ambrose PG. Antimicrobial safety: focus on fluoroquinolones. Clin Infect Dis. 2005;41(Supplement_2):S144–S157. 15942881 10.1086/428055

[10] Mattappalil A, Mergenhagen KA. Neurotoxicity with antimicrobials in the elderly: a review. Clin Ther. 2014;36(11):1489–1511.e4. 1511 e1484 25450476 10.1016/j.clinthera.2014.09.020

[11] Bruniera FR, Ferreira FM, Saviolli LR, et al. The use of vancomycin with its therapeutic and adverse effects: a review. Eur Rev Med Pharmacol Sci. 2015;19:694–700. 25753888

[12] Velkov T, Dai C, Ciccotosto GD, et al. Polymyxins for CNS infections: Pharmacology and neurotoxicity. Pharmacol Ther. 2018;181:85–90. 28750947 10.1016/j.pharmthera.2017.07.012

[13] Mani LY, Kissling S, Viceic D, et al. Intermittent hemodialysis treatment in cefepime-induced neurotoxicity: case report, pharmacokinetic modeling, and review of the literature. Hemodial Int. 2015;19(2):333–343. 25052578 10.1111/hdi.12198

[14] Neves PD, Freitas FM, Kojima CA, et al. Piperacillin/tazobactam-induced neurotoxicity in a hemodialysis patient: a case report. Hemodial Int. 2015;19(1):143–145. 25098503 10.1111/hdi.12194

[15] Zhang J, Huang C, Li H, et al. Antibiotic-induced neurotoxicity in dialysis patients: a retrospective study. Ren Fail. 2013;35(6):901–905. 23725229 10.3109/0886022X.2013.794684

[16] Nakagawa R, Sato K, Uesaka Y, et al. Cefepime-induced encephalopathy in end-stage renal disease patients. J Neurol Sci. 2017;376:123–128. 28431597 10.1016/j.jns.2017.03.018

[17] Lindsay H, Gruner S, Brackett J. Cefepime-Induced neurotoxicity despite dose adjustment for renal disease: a brief report and review of the literature. J Pediatric Infect Dis Soc. 2017;6(2):199–201. 27147713 10.1093/jpids/piw022

[18] Chow KM, Szeto CC, Hui AC, et al. Mechanisms of antibiotic neurotoxicity in renal failure. Int J Antimicrob Agents. 2004;23(3):213–217. 15164960 10.1016/j.ijantimicag.2003.11.004

[19] Pea F, Viale P, Pavan F, et al. Pharmacokinetic considerations for antimicrobial therapy in patients receiving renal replacement therapy. Clin Pharmacokinet. 2007;46:997–1038. 18027987 10.2165/00003088-200746120-00003

[20] Durand-Maugard C, Lemaire-Hurtel AS, Gras-Champel V, et al. Blood and CSF monitoring of cefepime-induced neurotoxicity: nine case reports. J Antimicrob Chemother. 2012;67(5):1297–1299. 22298349 10.1093/jac/dks012

[21] Chaibi K, Chaussard M, Soussi S, et al. Not all beta-lactams are equal regarding neurotoxicity. Crit Care. 2016;20(1):350. 27784324 10.1186/s13054-016-1522-z PMC5081676

[22] Sugimoto M, Uchida I, Mashimo T, et al. Evidence for the involvement of GABA(A) receptor blockade in convulsions induced by cephalosporins. Neuropharmacology. 2003;45(3):304–314. 12871648 10.1016/s0028-3908(03)00188-6

[23] Lager E, Nilsson J, Ostergaard Nielsen E, et al. Affinity of 3-acyl substituted 4-quinolones at the benzodiazepine site of GABA(A) receptors. Bioorg Med Chem. 2008;16(14):6936–6948. 18541432 10.1016/j.bmc.2008.05.049

[24] Lager E, Andersson P, Nilsson J, et al. 4-quinolone derivatives: high-affinity ligands at the benzodiazepine site of brain GABA a receptors. synthesis, pharmacology, and pharmacophore modeling. J Med Chem. 2006;49(8):2526–2533. 16610795 10.1021/jm058057p

[25] Johnstone TB, Hogenkamp DJ, Coyne L, et al. Modifying quinolone antibiotics yields new anxiolytics. Nat Med. 2004;10(1):31–32. 14647497 10.1038/nm967

[26] Davenport A. Measuring residual renal function for hemodialysis adequacy: is there an easier option? Hemodial Int. 2017;21 Suppl 2:S41–S46. 29064172 10.1111/hdi.12592

[27] Szeto CC, Li PK, Johnson DW, et al. ISPD Catheter-Related infection recommendations: 2017 update. Perit Dial Int. 2017;37(2):141–154. 28360365 10.3747/pdi.2016.00120

[28] Carvalho LAC, Correia MDL, Ferreira RC, et al. Accuracy of delirium risk factors in adult intensive care unit patients. Rev Esc Enferm USP. 2022;56:e20210222. 34989391 10.1590/1980-220X-REEUSP-2021-0222 PMC10184754

